# The Cause of Pellagra and Treatment Suggestions

**Published:** 1916-07

**Authors:** B. W. Page

**Affiliations:** Lumberton, N. C.


### The Cause of Pellagra and Treatment Suggestions.

BY B. W. PAGE, A. B., M. D., LUMBERTON, N. C.

The cause of pellagra is not yet settled to the satisfaction of the medical profession, and the more one reads the many theories advanced as to its etiology the more at a loss he becomes as to what is the correct answer. The majority sentiment among physicians who have had experience in treating the disease seems to be in favor of the germ theory, while many excellent laboratory men still hold to the dietetic theory as to etiology. In my opinion, as I have stated many times before, the source of all symptoms originate from an infected alimentary canal.

While making a large number of examinations of feces for various intestinal parasites, I have observed in pellagrins a peculiar organism which at first seemed to be a spirochâete or spirillum whose motility was of a corkscrew character. In fresh specimens they were almost constantly present, while in old specimens they were more often absent. In all specimens of pellagrins examined, a small, motile, cell-like body was present. In the majority of specimens a non-motile, beaded bacillus was observed. After repeated examinations I was convinced that all three forms are one and the same organism. This spore-producing bacillus invades pellagrin stools and causes them to present a different microscopic appearance from all other stools. A tentative diagnosis of the disease has been made from such findings regardless of clinical symptoms. Definite symptoms usually appear later unless the patient is carefully treated.

Attempts at cultivation seemed at first negative because the organism so often develops as a coccus, but by further study in pure culture and by transference to sterile feces as a medium all forms were soon observed. Later I was able to find an agglutinin which aids greatly in isolating the bacillus from feces. The organism is easily stained with watery solutions of Loeffler's methylene blue, with dilute carbol fuchsin and rather poorly with Gram's stain. The bacillus grows readily on the usual culture media at 37° C. and at room temperature. The growth is more luxuriant in a media containing carbohydrates.

*Corn Agar Plate.*—A twenty-four-hour colony measures from 1 to 2 millimeters in diameter. The surface is convex. The borders are smooth. The color is whitish gray. Forty-eight-hour colonies are umbonate and measure 3 millimeters in diameter.

*Corn Agar Slant.*—At twenty-four hours the growth is abundant. Form, aborescent; elevation, raised; lester, glistening; topography, smooth; optical characters, opaque; chromogenesis, grayish with age; odor, slight and apparently characteristic of the organism; consistency, a tendency to be slimy; medium, not effected.

*Potato.*—The twenty-four-hour growth is abundant. Surface rough and pitted. At the end of three days at incubator temperature the surface takes on a light tan color and growth covering surface has an areolate appearance. Luster dull.

The bacillus is aerobic and slightly facultative anaerobic; non-gas producing; liquefies gelatin; does not ferment dextrose. laetose, saccharose or maltose; peptonizes milk; slight pellicle formation on bouillon. While these characteristics conform in many respects to those of organisms of the subtilis mesentericus group, the organism differs from those of this group in staining, constant presence, growth on potato, pathogenicity, and serological reaction.

The bacillus has been isolated from pellagrin feces and grown in pure culture twenty-six different times, while ten attempts at cultivation from feces of non-pellagrins were negative. Care was taken to discard anyone as normal who had had in times past any suspicious symptoms of pellagra. After apparent immunity to the disease is established the bacilli seem to become normal inhabitants of the intestinal tract.

Experimental study of the organism has been made on mice with some degree of success. When administered by the alimentary canal the bacilli produce a toxin that causes symptoms apparently similar to those of pellagra. In the absence of dermatitis a definite diagnosis of the disease could not be made. The mice all died in twenty-four to ninety-six hours. The bacilli were present in all the organs after death. Pure cultures have been obtained.

The serum from blisters occurring on pellagrins furnishes a very satisfactory agglutinin for the bacilli. When examined in hanging drop in dilutions of from one to five or even from one to fifty the organisms are rendered non-motile and seem to clump in from three to thirty minutes. This reaction is of great assistance in identifying colonies and an aid in making a diagnosis of pellagra in doubtful cases, since the serum from any patient serves as a serological test for the bacilli wherever found, from infected mouse or man. I have not yet found other intestinal bacteria that respond to this test. Serum from artificial blisters of healthy individuals has no agglutinative power for the organism in question. The value of the serum as an agglutinin seems to vary with the toxemia of the pellagrin. The less toxic the patient the less dilution of serum from the artificial blister required for the reaction.

It appears to me that pellagra spreads as an infectious disease. The history of the disease in the South, in a given community or even in a home, seems to warrant such a conclusion. Following the first case in a home, other members of the family often become infected if the sanitary conditions are poor. This idea has been confirmed further by an accidental exposure of a laboratory assistant who developed the disease. Before exposure, both symptoms and bacilli were absent. The bacilli seem to be spread by hand and by flies.

In the prevention of pellagra, diet seems to be as important as it is in the prevention of tuberculosis; in the treatment of pellagra, it is more valuable. In addition to the toxemia produced by the specific infection, the etiological agent seems to decompose certain carbohydrates such as cane syrup, further increasing the severity of the disease.

After administering various intestinal antiseptics to pellagrins, I have found none that destroys the organism in question. As a symptomatic treatment, a few drugs are very important, and should be given freely. Of these, arsenic, sodium sulphoichthyolate, picric acid and milk of magnesia are recommended.

All cases of pellagra should be examined very carefully for uncinariasis, amebiasis and other parasitic diseases with which pellagra is so often complicated.

[In this number appears a scholarly paper by Dr. Page, of Lumberton, N. C., in which he describes an organism which he thinks may be a cause of pellagra. If his observations should be borne out by a sufficient number of observers, his name will become famous. Unfortunately the organism (?) he describes is so polymorphous that one cannot but remember any slide made from any specimen of feces in student days. Everything was there.

And an organism that is not satisfied with leading a double life but has to be at different stages of development a spirochete or spirillum, a motile, cell-like body, a non-motile bacillus, aerobic and possibly anaerobic, a coccus and a spore must certainly bring identification papers with it.

But don't mind me, Brother Page; if you are right, you are a great man; keep digging and get a moving picture of that bug.—EDITOR.]

---

As a result of a talk on pellagra by Dr. Wilmer L. Allison before the Fort Worth Doctors' Luncheon Club recently, it was unanimously decided to take up the question of forming a nucleus in Fort Worth for a State commission to investigate cases of pellagra and carry on research work. Chairman T. M. Jeter was authorized to appoint a committee of five physicians to begin a campaign for funds to support the work until such time as the State took the matter up and financed it.

---

In his vital statistics report for the month of May, Dr. Charles Tarver, City Health Officer of Eagle Pass, says that that city is still in the lead as the healthiest town in Texas. The report says there were fourteen deaths in the city during the month, thirteen being Mexicans and one American. The American was a woman 69 years old, and had gone to Eagle Pass several months before in the advanced stages of an illness. This was the first American death in Eagle Pass during the year and Eagle Pass has a population of three thousand Americans.

In this statement, Dr. Tarver says that the record up to the present time shows a decrease of fully 50 per cent in the death rate. He claims that the low death rate this year is due to the proper screening of foods offered for sale and better hygienic conditions in general.

